# Aging affects artemisinin synthesis in *Artemisia annua*

**DOI:** 10.1038/s41598-021-90807-1

**Published:** 2021-05-28

**Authors:** Jiao Li, Xiao-Hui Chu, Xiao-Yu Wang, Bao-Min Feng, Zong-Xia Yu

**Affiliations:** College of Life Sciences and Biotechnology, Dalian University, 10 Xuefu Street, Dalian Economic and Technological Development Zone, Dalian, 116622 Liaoning China

**Keywords:** Gene regulation, Sequencing, Transcriptomics, Plant molecular biology

## Abstract

Artemisinin (ART) is the most effective component in malaria treatment, however, the extremely low content restricts its clinical application. Therefore, it is urgent to increase the yield of ART. ART gradually accumulates with aging, small RNA (sRNA) and transcriptome analysis were applied on the leaves of 2-week-old (2 w) and 3-month-old (3 m) *A. annua* respectively. Among all the annotated sRNAs, 125 were upregulated and 128 downregulated in the 3 m sample compared to the 2 w one. Whereas 2183 genes were upregulated and 2156 downregulated. Notably, the level of *miR156* and several annotated *miRNA*s gradually decreased while *SPL*s increased. In addition, the genes on ART biosynthesis pathway were significantly upregulated including *ADS*, *CYP71AV1*, *ADH1*, *DBR2* and *ALDH1*, and so were the positive transcription factors like AaERF1, AaORA and AaWRKY1 indicating that age influences the ART biosynthesis by activating the expression of the synthesizing genes as well as positive transcription factors. This study contributes to reveal the regulatory effects of age on ART biosynthesis both in sRNA and transcription levels.

## Introduction

Malaria still threatens global human health which infects 214 million people and causes 430,000 deaths every year according to the world health organization's report. Scientists have been always working on preventing the spread of malaria since the 1940s, but malaria remains a tough problem in sub-Saharan Africa^1^. Artemisinin (ART) were first reported by Chinese scientists in Chinese science bulletin in 1977. Nowadays ART-based combination therapies (ACTs) is the primary treatment for malaria, especially the falciparum ones. In 2015, Dr. Youyou Tu won the Nobel Prize for her great contribution to the discovery of ART and its anti-malaria efficacy. In addition, ART and its derivatives also have anti-cancer, anti-parasite and other pharmacological effects^2^. Despite the huge market demand, the supply of ART is extremely insufficient for the following reasons. First, ART is mainly extracted from the wild *A. annua* plants, but the planting area and biomass of *A. annua* fluctuate greatly every year. Second, the content of ART in the wild *A. annua* is extremely low only 0.1% to 0.8% of dry weight^3^. Therefore, it is urgent to increase the yield of ART. Over-expression of the biosynthesis pathway genes, transcriptional regulation and blocking the competitive pathways have been applied to increase the content of ART in *A. annua*, however, how age regulates the biosynthesis of ART is still far been explored considering the fact that the content of ART accumulates with plant age and reaches maximum after bloom. The sRNA and transcriptome analysis will provide the differentially expressed genes related to age, which will aid to elucidate the link between age and ART biosynthesis.

We mainly focus on three key targets in transcriptome analysis. Firstly, *miR156* and its targeted *SPL* genes. *miR156* is the only reported age cue in plant, and it functions by targeting the *SPL* genes. Secondly, ART biosynthesis genes. ART is a sesquiterpene lactone with a specific endo-peroxide bridge, which is synthesized by isoprenoid metabolic pathway. FPP is catalyzed by ADS (amorpha-4,11-diene synthase) to form amorpha-4,11-diene; then artemisinic alcohol(AAOH) is formed under the catalysis of a cytochrome P450 monooxygenase CYP71AV1 (cytochrome P450-dependent hydroxylase); artemisinic aldehyde(AAA) is formed under the combined action of CYP71AV1 and ADH1 (alcohol dehydrogenase 1); DBR2 (artemisinic aldehyde Δ11(13) reductase) converts artemisinic aldehyde to dihydroartemisinic aldehyde (DHAAA), which is further oxidized to dihydroartemisinic acid(DHAA) by ALDH1^4^. Thirdly, transcription factors. Many transcription factors have been reported to positively regulate the ART biosynthesis by activating the expression of pathway genes. AaWRKY1 activates the transcription of *CYP71AV1*^5^. AaORA regulates the expression of *ADS*, *CYP71AV1* and *DBR2*^6^. AaERF1, AaERF2, TAR1, AabZIP1 and AabHLH1 specifically increase the expression of *ADS* and *CYP71AV1*^4,7–9^. AaNAC1 upregulates the expression of *ADS* and *CYP71AV1*^10^. AaMYC2, an activator of jasmonic acid (JA) signal pathway can improve the expression levels of *CYP71AV1* and *DBR2*^11^. This transcriptome study will aid to construct the link between age and ART biosynthesis by analyzing these above mentioned key targets.

## Results

### Procession and statistics of the raw data

**Figure 1:**
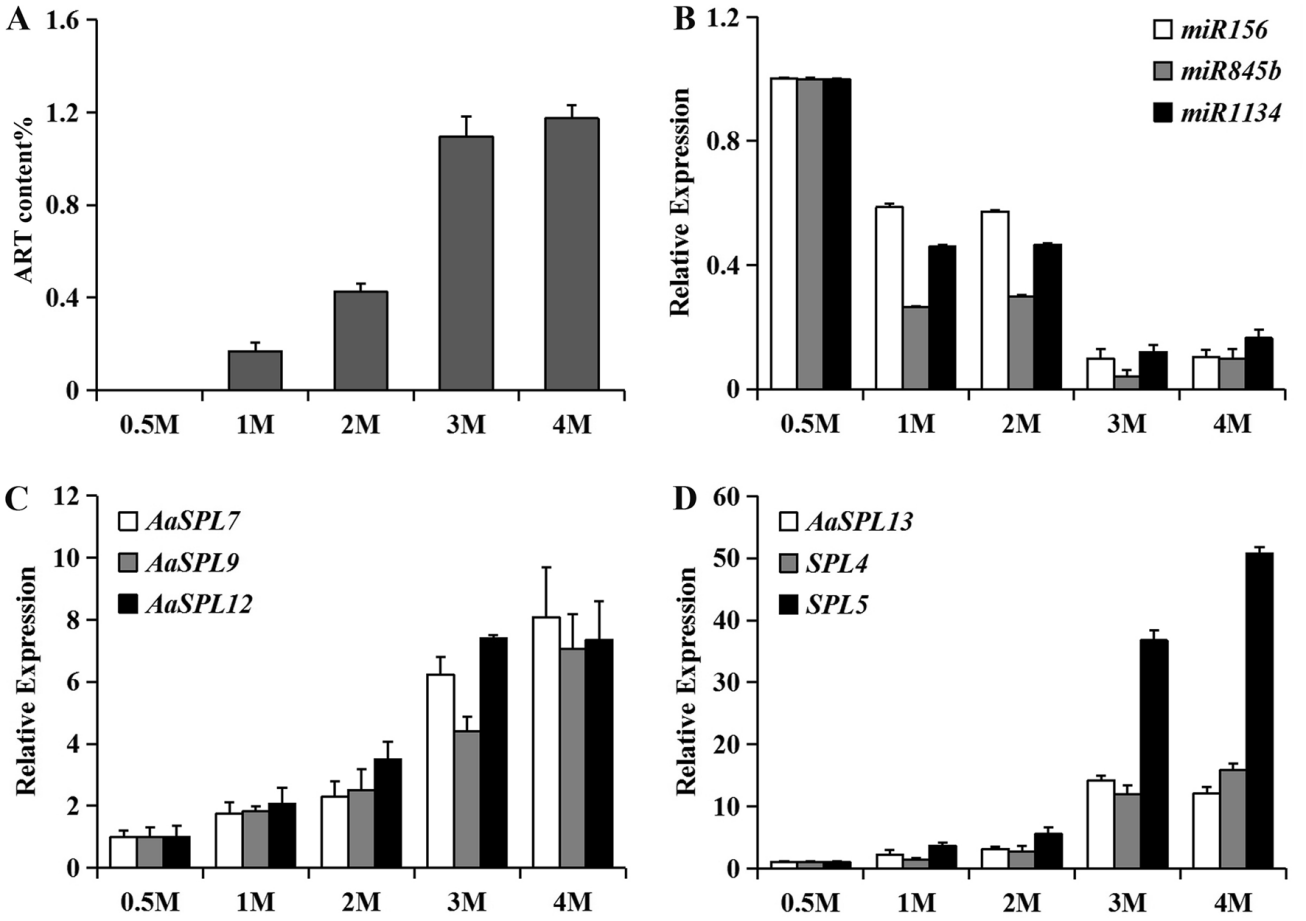
ART content and expression of *miRNA* and *SPL*s accumulated with age. (**A**) ART content in leaves of 0.5, 1, 2, 3 and 4-month old *A. annua*. The ART content was compared to the dry weight of the sample to gain the percent (%). (**B**–**D**) qRT-PCR analysis of miRNAs and genes in in leaves of *A. annua* in different age*. miR156*, *AaSPL7*, *AaSPL9*, *AaSPL12* and *AaSPL13* were reported^18,19^, *miR845b*, *miR1134*, *SPL4 and SPL5* were found from the sequencing data.

In order to address the link between age and artemisinin (ART) biosynthesis, the ART content in the leaves of *A. annua* in different age were analyzed. The ART content increased gradually with age though it was undetectable in half month (0.5 M) and the rate lowed down in 3 months (3 m) (Fig. 1A). Thus the leaves of 2-week-old (2 w) and 3-month-old (3 m) *A. annua* were sampled for RNA sequencing respectively, and named as S2 w and S3 m. The leaves of at least three individual lines of 2 w and 3 m mixed.

The original data from the sequencing contains low quality sequences with joints. In order to ensure the quality of information analysis, the original data must be filtered to obtain clean data, and subsequent analysis is based on clean data. The quality assessment of sample sequencing output data is shown in the following table (Table 1). Then the Bowtie 2 v2.1.0 was used to map the reads of each sample to the reference of *A. annua* genome^12–14^, and the parameters are defaulted, only 10.63% to 11.32% of the sRNA could mapped while 43.63% to 44.09% of the transcriptome could uniquely mapped to the reference genome (Table 2).

**Table 1:** Statistics of the sequencing data.

Items	sRNA		Items	Transcriptome	
	S2 w	S3 m		S2 w	S3 m
Total sRNA	19,521,529	25,255,008	Raw reads	31,646,158	19,080,382
Known miRNA	16,481	15,957	Clean reads	29,192,760	17,681,871
Other anno sRNA	702	757	Read 1 Q30	98.43%	98.39%
Unanno sRNA	2,112,083	2,559,637	Read 2 Q30	98.00%	98.09%

Known miRNA: The sRNAs annotated by plant miRBase database.

Other anno sRNA: annotated sRNAs other than rRNA, tRNA, sRNA, snRNA and miRNA.

Unanno sRNA: unannotated sRNAs.

Raw Reads: The number of sequencing sequences of each document is counted in four action units by counting the original sequence data.

Clean reads: The calculation method was the same as Raw Reads, but the statistical files were filtered data, and subsequent analysis was based on this.

Q20, Q30: The percentages of bases with phred value greater than 20 and 30 in the total bases were calculated respectively.

**Table 2:** Statistics of the clean reads mapped to the genome.

Items	sRNA		transcriptome	
	S2 w	S3 m	S2 w	S3 m
Total reads	19,521,529	25,255,008	29,192,760	17,681,871
Mapped reads	2,210,537	2,683,853	24,634,525	14,447,092
Unique mapped reads	–	–	12,871,457	7,714,583
Mapped ratio	11.32%	10.63%	84.39%	81.71%
Unique mapped ratio	–	–	44.09%	43.63%

Total reads: The total number of clean reads obtained by sequencing the sample.

Mapped reads and Mapped ratio: The number and the ratio of the clean reads mapped to the reference genome.

Unique mapped reads and Unique mapped ratio: The number and the ratio of the unique reads mapped to the reference genome.

− no statistics.

### Several miRNAs and ART biosynthesis related genes accumulated with age

To estimate the differentially expressed genes (DEGs) between different ages, the concept of RPKM was introduced^15^. The MARS (MA-plot-based method with Random sampling model) model in DEGseq v1.20.0 package was used to analyze the differences^16^. The difference of gene expression is considered to be significant in the condition of |Fold change| > 2, FDR (q value) < 0.001, and at least one sample RPKM > 20. Statistical analysis was conducted on all the genes with significant expression difference between the S3m and S2w samples (Table 3).

**Table 3:** Statistics of the genes expression with significant difference.

DEGs S3 m vs. S2 w	sRNA		Transcriptome	
	Amount	Ratio (%)	Amount	Ratio (%)
Upregualted genes	129	50.79	2156	49.69
Downregualted genes	125	49.21	2183	50.31

**Figure 2:**
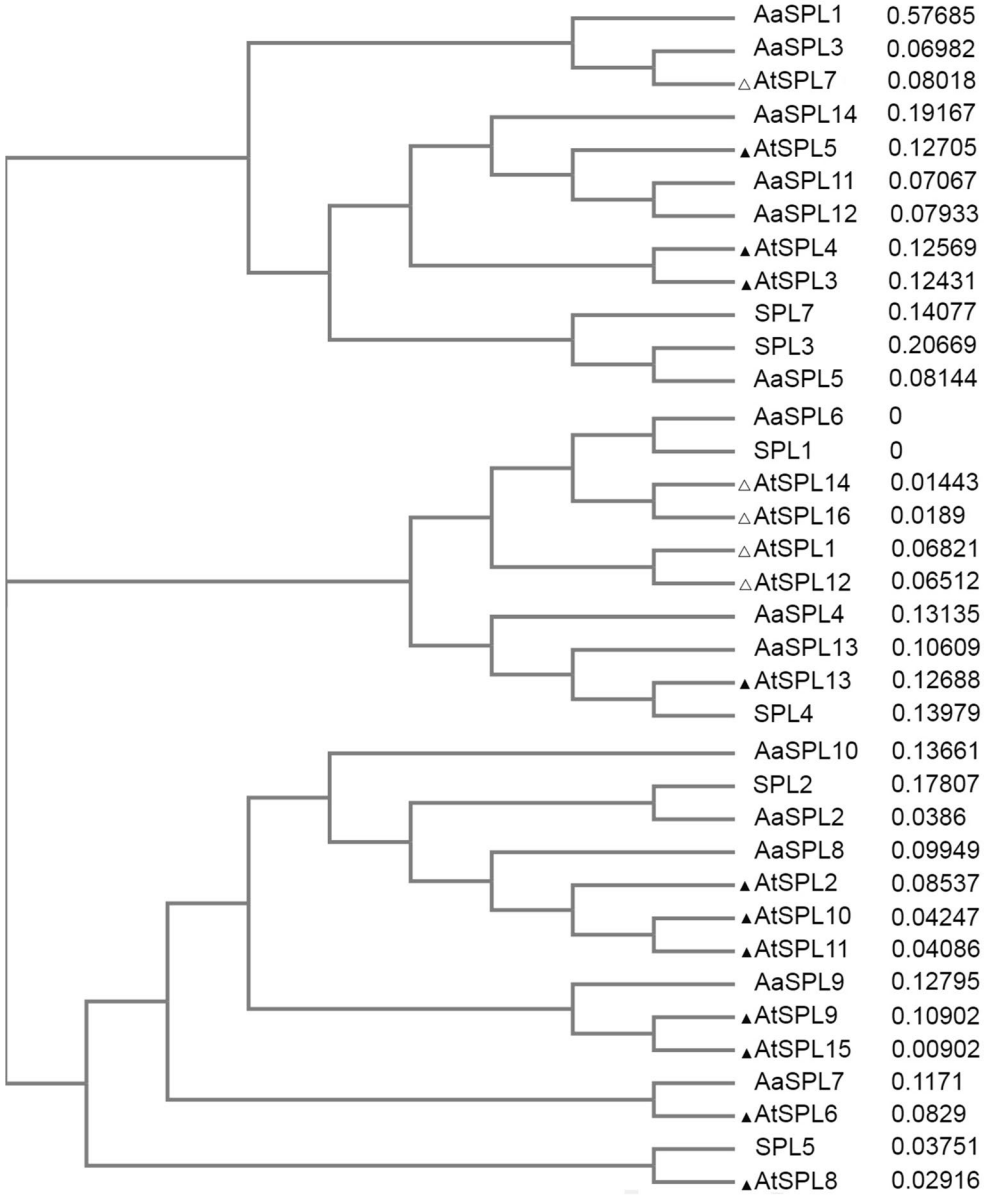
Phylogenetic tree of the SBP domain of SPLs from *A. annua*.and *Arabidopsis*. The tree was constructed using Clustal Omega (https://www.ebi.ac.uk/Tools/msa/clustalo/) with Neighbor-Joining method. The black triangle (▲) or blank triangle (△) indicates miR156 targeted or non-targeted *Arabidopsis* SPLs respectively.

The expression levels of reported miRNA and predicted novel miRNA are calculated between these two samples, 254 DEGs were obtained among which 50.79% were upregualted and 49.21% downregulated (Table 3). It has been predicted that miRNAs belong to miR414 and miR1310 families may target genes in ART biosynthesis like HMG-CoA reductase (HMGR), amorpha-4,11-diene synthase (ADS), farnesyl pyrophosphate synthase (FPS) and cytochrome P450^17^, unfortunately these miRNAs have not been detected in our sequence data. However, there were still six known miRNAs downregulated significantly (Supplement Fig. 1A). In addition, miR156 which is conserved in plant kingdom and abundant in the junior plant is also not detected in the sequence data, however, the expression of some predicted *SPL* genes show vigorous varieties (Supplement Fig. 1B). Luckily *miR156* and several *AaSPL*s have been reported by other groups^18,19^. The *SPL* genes could be targeted or non-targeted by miR156, the reported *AaSPL*s and those from the our RNAseq data were aligned with *SPL*s from *Arabidopsis* (https://www.ebi.ac.uk/Tools/msa/clustalo/), *AaSPL7*, *AaSPL9*, *AaSPL12*, *AaSPL13*, *SPL4* and *SPL5* showing more similarities to miR156-target-SPLs in *Arabidopsis* were further confirmed (Fig. 2). The level of miR156^18^ and two novel miRNAs, miR845b and miR1134 declined with age, and the picked SPLs^19^ enhanced to varying degrees (Fig. 1B–D). Indicating miR156 and targeted SPLs works as age cue in *A. annua* too. Because of the low abundance of miR156 in *A. annua*^18^, there may be other miRNAs to complement its role.

**Figure 3:**
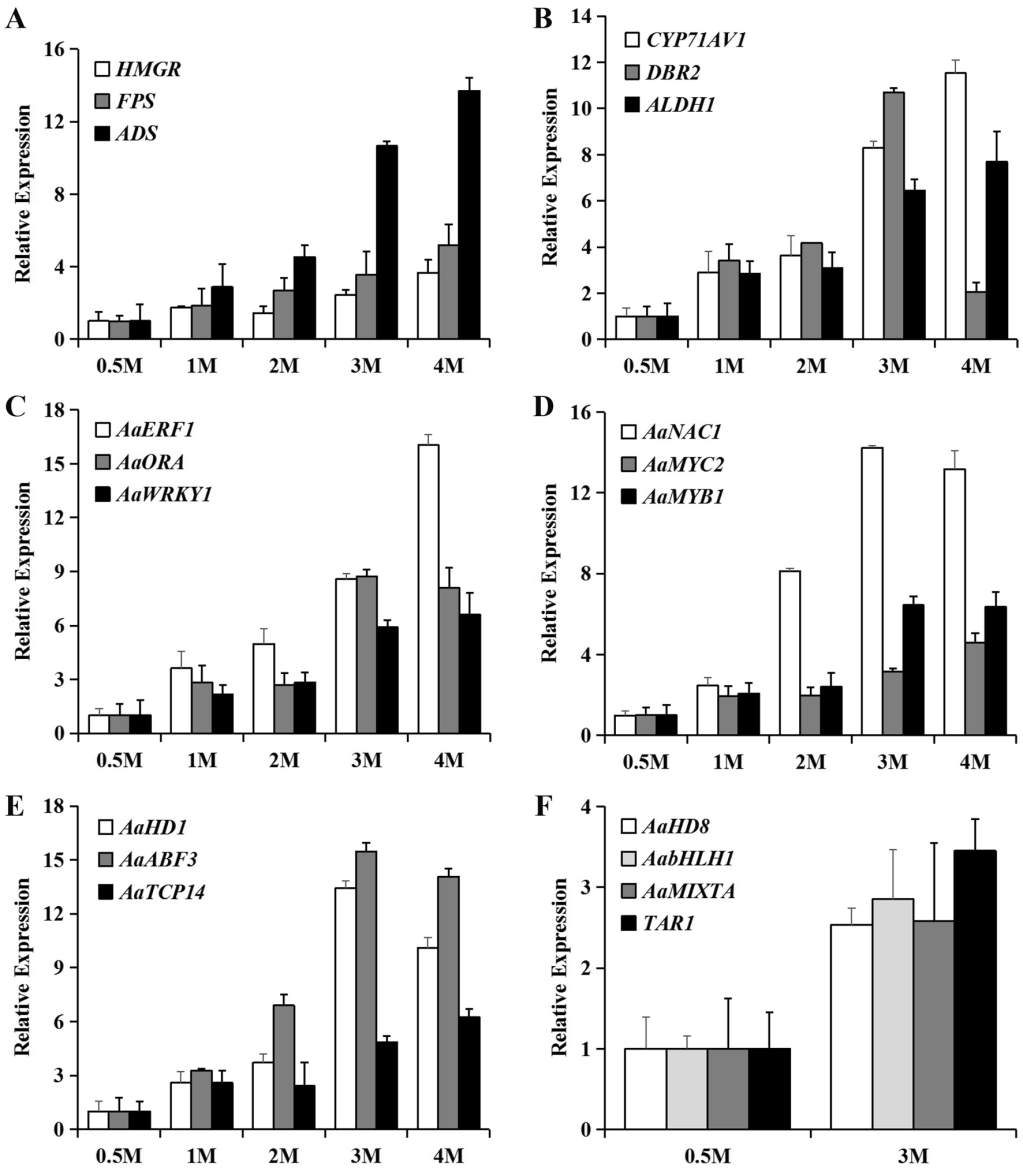
Expression of ART biosynthetic genes and transcription factors increased with age. qRT-PCR analysis of ART biosynthetic genes (**A**, **B**) and transcription factors (**C**–**F**) in leaves of 0.5, 1, 2, 3 and 4-month old *A. annua.*

There are 4339 differentially expressed genes in transcriptome, of which 49.69% were upregulated and 50.31% were downregulated. The transcription levels of the key genes in the biosynthesis of ART including *ADS*, *CYP71AV1*, *ADH1*, *DBR2* and *ALDH1* were all increased in S3m compared to S2w (Supplement Fig. 1C), which was in accordance with the phenomenon that ART accumulated with age. In addition, the positive regulatory transcription factor AaWRKY1 was obviously upregulated, and several other genes identical to the reported positive transcription factors like AaERF1, AaMYC2, AabHLH1 and AabZIP1 were upregulated as well (Supplement Fig. 1D). ART biosynthetic genes and reported regulation factors were further analyzed by qRT-PCR. Expression of most genes increased along with age, but the level of DBR2 descended in the four-month plant while AaHD8, AabHLH1, AaMIXTA and TAR1 didn’t change much (Fig. 3). These transcription factors may construct a link between age and the ART biosynthesis which needs further detection.

### Process involved in growth and metabolism are enriched with age

**Figure 4:**
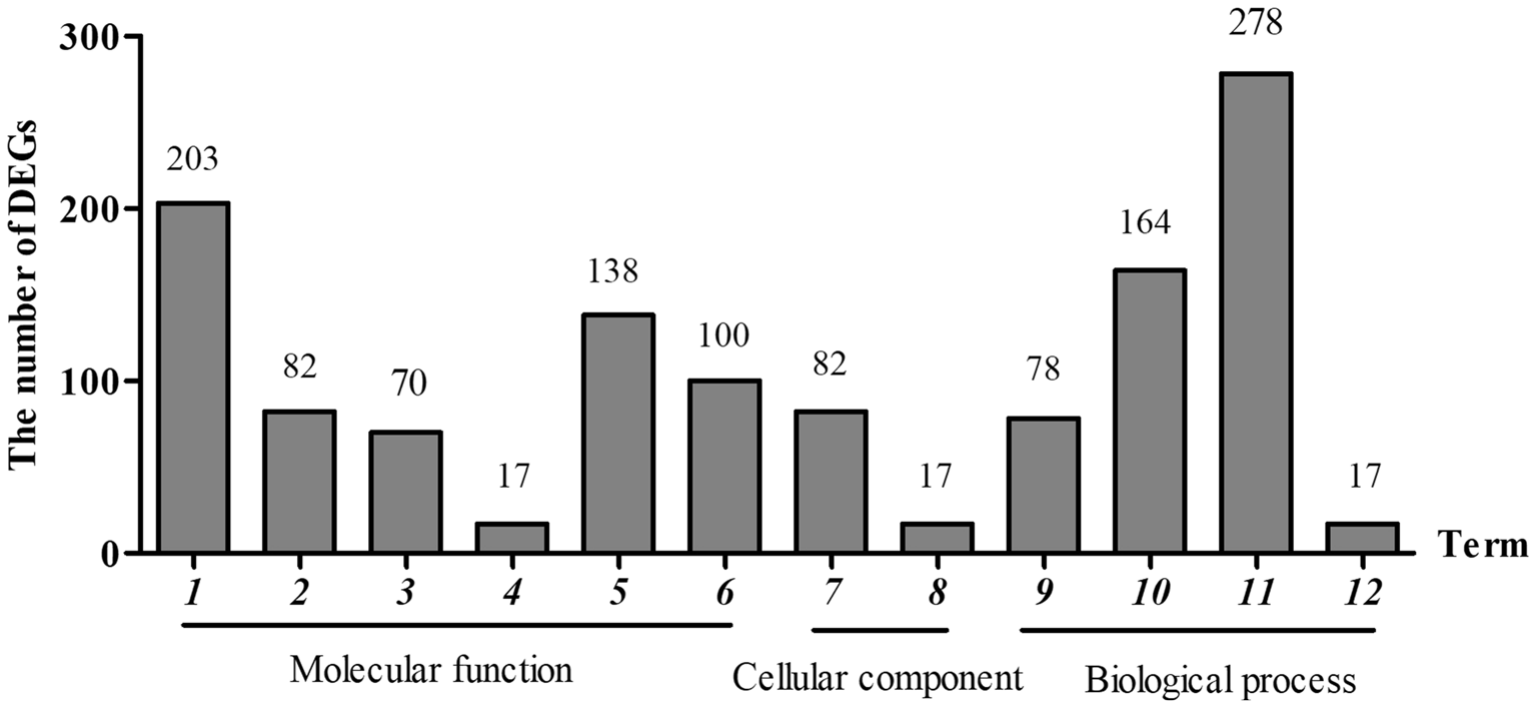
GO enrichment of DEGs. 1. DNA binding. 2. Structural constituent of ribosome. 3. Hydrolase activity. 4. Xyloglucan: xyloglucosyl transferase activity. 5. Oxidoreductase activity. 6. Transcription factor activity; sequence-specific binding. 7. Ribosome. 8. Apoplast. 9. Translational elongation. 10. Regulation. 11. oxidation–reduction process. 12. Cellular glucan metabolic process.

To gain functional annotations of the genes involved in age-related ART accumulation, GO analysis was carried out for DEGs. GO enrichment analysis using GOseq showed that the FDR (q value) value was less than 0.05 and the GO term was regarded as the enrichment term^20^. 1,237 DEGs were annoted in the GO analysis. Both the upregulated and downregulated genes of DEGs of *A. annua*’s transcriptome were classified into three functions: Biological Process (BP), Cellular Component (CC), and Molecular Function (MF). Many terms of plant metabolites catalysis process were significantly enriched in these biological processes (FDR ≤ 0.05 or P-value ≤ 0.05). Among the upregulated genes 1770 were functional annotated including 133 terms for BP, 27 terms for CC, and 281 terms for MF. Photosynthesis, metabolic process including the biosynthetic and catabolic process, morphogenesis of shoot, and developmental process of embryo and seed are included in BP. Chloroplast, plasmids and NAD(P)H dehydrogenase complex are involved in CC. While ribosome construction, activity of transcription factor and binding of RNA polymerase and chlorophyll are contained in MF. While in the downregulated genes, 2023 were annotated with functions, comprising of 112 terms in BP, 37 in CC, and 209 in MF. GO enrichment histogram of DEGs can directly reflect the distribution of the number of differentially enriched genes in each GO entry. 12 GO items with the most significant enrichment were selected for display (Table 4), and the majority of the DEGs are involved in DNA binding, redox enzyme activity, transcription factor activity (sequence-specific binding), transcriptional regulation and redox process are shown (Fig. 4).

**Table 4:** 12 GO items with the most significant enrichment.

GO accession	Term	Ontology	Sample number	Background number	Over represented pvalue	BH adjust
GO:0003677	DNA binding	Molecular function	203	1513	5.68E−12	5.49E−09
GO:0005840	ribosome	Cellular component	82	491	9.31E−12	5.49E−09
GO:0003735	Structural constituent of ribosome	Molecular function	82	503	3.63E−11	1.42E−08
GO:0006414	Translational elongation	Biological process	78	490	3.40E−10	1.00E−07
GO:0,006,355	Regulation of transcription	Biological process	164	1278	5.30E−08	1.25E−05
GO:0004553	Hydrolase activity	Molecular function	70	419	1.13E−07	2.22E−05
GO:0055114	Oxidation–reduction process	Biological process	278	2397	1.13–06	0.000167237
GO:0006073	Cellular glucan metabolic process	Biological process	17	55	2.14E−06	0.000229502
GO:0016762	Xyloglucan:xyloglucosyl transferase activity	Molecular function	17	55	2.14E−06	0.000229502
GO:0048046	Apoplast	Cellular component	17	55	2.14E−06	0.000229502
GO:0016491	Oxidoreductase activity	Molecular function	138	1065	4.07E−06	0.000399892
GO:0003705	Transcription factor activity;sequence-specific binding	Molecular function	100	699	1.91E−07	3.22E−05

### Biochemical metabolic pathways and signal transduction pathways concentrated following age

**Figure 5:**
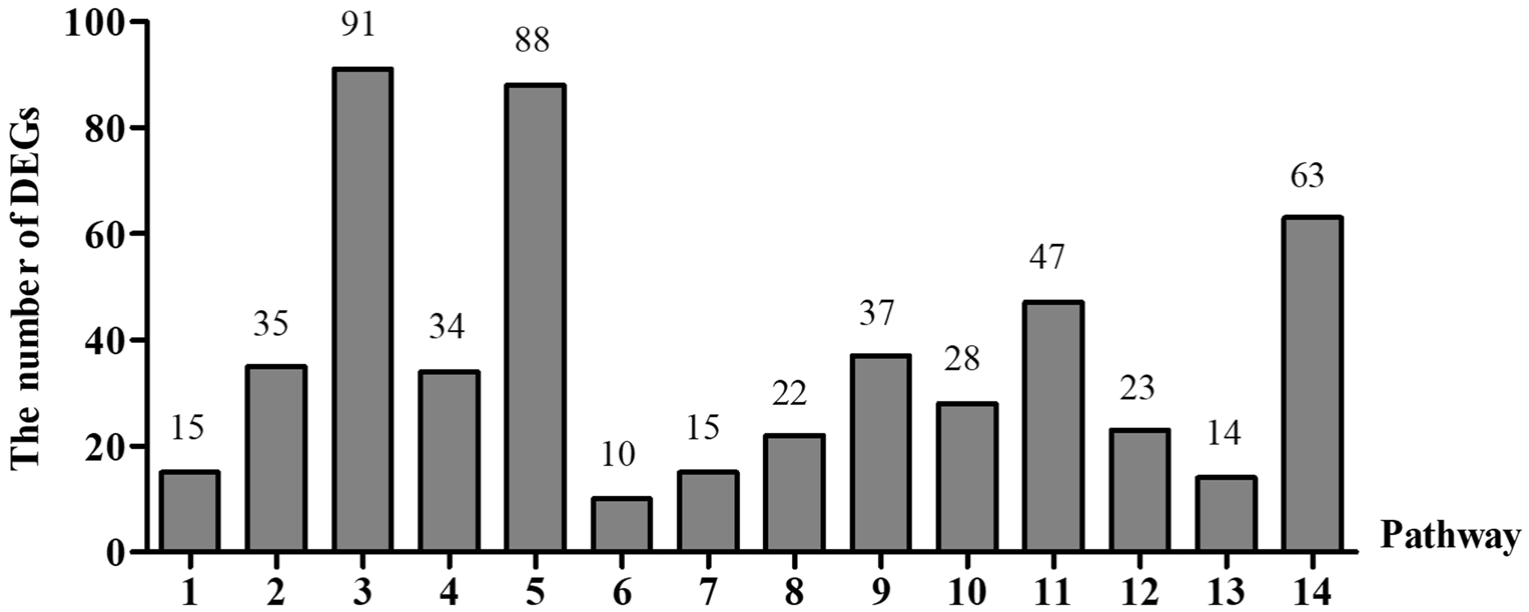
KEGG enrichment of DEGs. 1. Alpha-Linolenic acid metabolism. 2. Carbon fixation in photosynthetic organisms. 3. Ribosome. 4. Glyoxylate and dicarboxylate metabolism. 5. Carbon metabolism. 6. Cell cycle—Caulobacter. 7. Cyanoamino acid metabolism. 8. Methane metabolism. 9. Glycolysis/gluconeogenesis. 10. Cysteine and methionine metabolism. 11. Plant hormone signal transduction. 12. Glycine, serine and threonine metabolism. 13. Photosynthesis—antenna proteins. 14. Biosynthesis of amino acids.

Generally genes coordinate with each other to perform their biological functions. KEGG^21^, pathway analysis was conducted to determine the main biochemical metabolic pathways and signal transduction pathways in age related ART biosynthesis. Pathway with FDR (q value) less than 0.05 was defined as pathway with significant enrichment (Table 5). A total of 522 DEGs were annotated in KEGG classification. 334 upregulated DEGs with functional annotations were involved in 174 metabolic pathways while that was 220 metabolic pathways in 458 downregulated DEGs. Carbon metabolism, plant hormone signal transduction and biosynthesis of amino acids are the three significantly fluctuated ones in both upregualted and downregulated pathways (Fig. 5), which indicates their conserved and major roles in the aging process of artemisia. The results suggesting that the higher metabolic pathways associated with ART biosynthesis were ribosome, carbon metabolism, and plant hormone signal transduction .

**Table 5:** Enrichment results of KEGG of DEGs.

Pathway	ID	Sample number	Background number	Over represented p value	BH adjust
Alpha-linolenic acid metabolism	ko00592	15	38	5.69E−05	1.91E−03
Carbon fixation in photosynthetic organisms	ko00710	35	88	5.57E−10	8.42E−08
Ribosome	ko03010	91	381	8.15E−09	6.15E−07
Glyoxylate and dicarboxylate metabolism	ko00630	34	91	6.76E−09	6.15E−07
Carbon metabolism	ko01200	88	308	5.38E−13	1.62E−10
Cell cycle—caulobacter	ko04112	10	24	0.000604	1.52E−02
Cyanoamino acid metabolism	ko00460	15	38	5.69E−05	1.91E−03
Methane metabolism	ko00680	22	66	2.68E−05	1.16E−03
Glycolysis/gluconeogenesis	ko00010	37	153	0.0002	6.04E−03
Cysteine and methionine metabolism	ko00270	28	117	0.001375	3.19E−02
Plant hormone signal transduction	ko04075	47	211	0.000242	6.63E−03
Glycine, serine and threonine metabolism	ko00260	23	93	0.002271	4.90E−02
Photosynthesis—antenna proteins	ko00196	14	27	2.08E−06	1.26E−04
Biosynthesis of amino acids	ko01230	63	279	1.36E−05	6.86E−04

## Discussion

ART is a sesquiterpene lactone compound extracted from sweet wormwood *A. annua*, which contains the specific endoperoxide bridge, and it is synthesized by isoprenoid metabolic pathway. *ADS*, *CYP71AV1*, *ADH1*, *DBR2* and *ALDH1*, as the key enzyme genes in the ART biosynthesis pathway, play an important role in ART biosynthesis. Overexpressing these genes is an important way of increasing ART content by genetic engineering. The transcripts of these genes are more abundant in the elder plants than the younger ones, which construct the link between ART biosynthesis and plant aging and provide the possibility of achieving higher ART production by harvesting the plants after blooming in addition with overexpressing enzyme genes.

Another way to improve ART yield is to overexpress the positive transcription factors of ART biosynthetic pathway enzymes. As we know transcription factors often simultaneously regulate the expression of more than one enzyme in the biosynthetic pathway, which could be a more effective way to increase ART content. Several transcription factors have been reported to elevate ART biosynthesis by targeting different enzyme genes on ART biosynthesis pathway, like AaWRKY1, AabZIP1, AabHLH1, AaMYC2, AaERF1 and AaERF2. Those positive regulators of ART synthesis amounted with plant age. Our transcriptome data imply more unknown transcription factors should be investigated especially these AP2/ERF ones, and this will further illuminate the way of enlarging ART production by combining these ways.

miR156 has been the only reported plant age cue, and it contributes to the anthocyanin biosynthesis in *Arabidopsis* and sesquiterpene biosynthesis in both *Arabidopsis* and *Patchouli* along with its critical roles in plant development^4,22^. The phenomenon that biosynthesis of ART mounts with age and is most vigorous after blooming indicating the effect of age on ART biosynthesis. Even though the miR156 is not detected in our small RNA sequencing data maybe because of the sequencing depth, selected samples or low abundance. Another group reported the miR156 sequence of *A. annua* with very low transcript level. Though miR156 gradually decreases while *SPL*s increases with age, whether other miRNAs or transcription factors play roles on aging and ART biosynthesis shall be further explored.

This study provide the molecular basis for the common sense that the aerial parts of *A. annua* are collected for ART extraction after blooming when both the biomass and ART content are high. The transcriptome data provide cues for key transcription factor mining for the regulation of ART biosynthesis. Meanwhile the small RNA sequencing data give some clues on investigation of *A. annua* aging and ART accumulation.

## Materials and methods

All experiments were conducted in accordance with relevant institutional, national, and international guidelines and legislation.

### Total RNA extraction and quality control

*A. annua* L. cv. QT was used in this paper. Total RNA was extracted from the leaves of two-week (2 w) and three-month (3 m) old *A. annua*.with Trizol (Invitrogen). The quality of total RNA was tested using 2100 Bioanalyzer. The qualified RNA samples were digested by DNaseI (TaKaRa, Japan) at 37 °C for 30 min.

### cDNA library construction and sequencing

DNase digested RNA was treated with Dynabeads Oligo (dT)25 kit (Life, USA) to get the purified mRNA. 100 ng purified mRNA was then treated with NEBNext Ultra RNA Library Prep Kit for Illumina (NEB, USA) to build the cDNA Library. The quantity of the cDNA library was further tested by Qubit quantification, 2% agarose gel electrophoresis detection and high-sensitivity DNA chip detection. 10 ng cDNA was used to cluster generation in cBot with TruSeq PE Cluster Kit (illumina, USA), and then bidirectional sequencing was performed in Illumina Hiseq 4000.

The transcripts were gained by referring to the whole genome sequencing data of *A. Annua*^14,23^, and then the differentially expressed genes (DEGs) were analyzed.

### Seperation of the small RNA (sRNA)

100 mg samples were first grinded into powder in liquid netrogen, and then the total RNA was extracted by Trizol kit (Invitrogen). The total RNA was seperated by 15% polyacrylamide gel electrophoresis, and the RNA located within 15-35nt by referring to the RNA TrackIt 10 bp DNA Ladder (Invitrogen) were purified as the sRNA.

### sRNA library construction and sequencing

The sRNA library was constructed using Truseq Small RNA Sample Preparation Kit (illumina), and the PCR products were seperated by 6% polyacrylamide gel electrophoresis and those located at 147 bp was purified as the sRNA cDNA library. The library was further amplified with TruSeq PE Cluster Kit (illumina, USA) and then sequenced by illumina Hiseq. After sequencing the clean data were gained by eliminating the low quanlity data, adaptors and contamination from the raw data, then the clean data were aligned and annotated with known rRNA, tRNA, nRNA, snoRNA and fragments of degraded mRNA in the database to eliminate the non-miRNA sequences. The unknown sRNA were further analyzed by novel miRNA software.

### Expression analysis

One microgram of total RNA was used for cDNA synthesis with oligo (dT) primers and M-MLV Reverse Transcriptase (Invitrogen) while miRNAs were inverse transcripted with miRNA 1^st^ strand cDNA synthesis kit (Accurate Biotechnology). Quantitative real-time RT-PCR was performed with SYBR-Green PCR Mastermix (Accurate Biotechnology). *A. annua* ACTIN (EU531837) and U6 (PWA96665) were used as internal references for mRNA and miRNA respectively.

### Chemical analysis

Contents of ART were determined by high-performance liquid chromatography (HPLC) as described^4^, and the standard substance of ART was bought form Sigma.

## Supplementary Information

Supplementary Legend.

Supplementary Figure S1.
