# Supplementary material for: Aging affects artemisinin synthesis in Artemisia annua
Source: Sci Rep. 2021 May 28;11:11297. doi: 10.1038/s41598-021-90807-1 (PMC8163859; doi:10.1038/s41598-021-90807-1)
Supplement: Supplementary file 1 — Supplementary Legend. [file 41598_2021_90807_MOESM1_ESM.doc]

**Supplement Fig. 1 Differential expression of *miRNA*, *SPL*s, ART biosynthetic genes and transcription factors shown in the sequencing data.**
