# Supplementary figures and images for: Aging affects artemisinin synthesis in Artemisia annua
Source: Sci Rep. 2021 May 28;11:11297. doi: 10.1038/s41598-021-90807-1 (PMC8163859; doi:10.1038/s41598-021-90807-1)

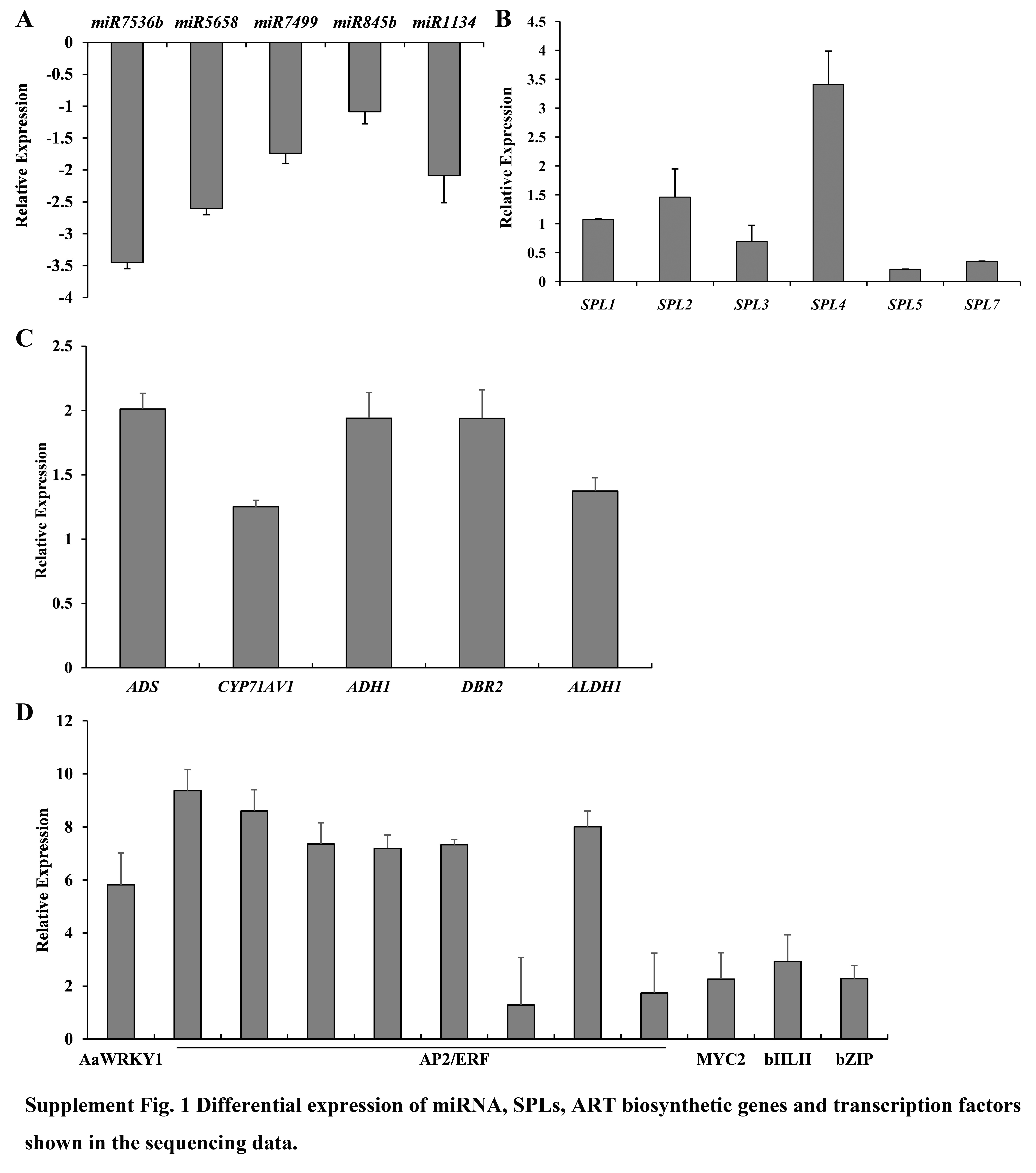

Supplement: Supplementary file 2 — Supplementary Figure S1. [file 41598_2021_90807_MOESM2_ESM.jpg]
